# Historical Building Monitoring Using an Energy-Efficient Scalable Wireless Sensor Network Architecture

**DOI:** 10.3390/s111110074

**Published:** 2011-10-25

**Authors:** Juan V. Capella, Angel Perles, Alberto Bonastre, Juan J. Serrano

**Affiliations:** ITACA Institute, Universitat Politècnica de València, 46022 Valencia, Spain; E-Mails: jcapella@disca.upv.es (J.V.C.); bonastre@disca.upv.es (A.B.); jserrano@disca.upv.es (J.J.S.)

**Keywords:** wireless sensor network, low-power nodes, multi-hop networks, termites sensor, moisture sensor

## Abstract

We present a set of novel low power wireless sensor nodes designed for monitoring wooden masterpieces and historical buildings, in order to perform an early detection of pests. Although our previous star-based system configuration has been in operation for more than 13 years, it does not scale well for sensorization of large buildings or when deploying hundreds of nodes. In this paper we demonstrate the feasibility of a cluster-based dynamic-tree hierarchical Wireless Sensor Network (WSN) architecture where realistic assumptions of radio frequency data transmission are applied to cluster construction, and a mix of heterogeneous nodes are used to minimize economic cost of the whole system and maximize power saving of the leaf nodes. Simulation results show that the specialization of a fraction of the nodes by providing better antennas and some energy harvesting techniques can dramatically extend the life of the entire WSN and reduce the cost of the whole system. A demonstration of the proposed architecture with a new routing protocol and applied to termite pest detection has been implemented on a set of new nodes and should last for about 10 years, but it provides better scalability, reliability and deployment properties.

## 1. Introduction

The field of wireless sensor networks is an emerging area of research that has been under intense study in recent years. These sensor networks represent a clear advance as regards practical future implementation, but most of usually proposed architectures face scalability problems when applied to our particular requirements.

Our problem involves the monitoring of wooden masterpieces and structures of heritage buildings. Given that in this environment maintenance is practically impossible, deployed nodes must work for years without operator intervention. These nodes were designed and developed with help from AIDIMA (Furniture, Wood and Packaging Technology Institute) and are being used for monitoring heritage wood structures and masterpieces. This system is currently in operation in the Valencia Cathedral (Spain).

Our wireless nodes are utilized for the ambient and pest monitoring of wood. Ambient monitoring is performed by measuring the relative ambient humidity and temperature to compute the equilibrium moisture content of the wood (EMC). Pests are detected using LEDs and light sensors which detect reflection variations when an insect such as a termite, ant, cockroach, *etc*. crosses the detector’s field. Most of the energy requirements of these nodes are invested in pest detection.

The installed implementation of the monitoring system is based on a star configuration, where nodes send their information to a sink. When a large number of nodes are required, the star configuration is unsuitable as it does not scale well; this is the case, say, if we need to implement such a system in a historical building, whose structure does not lend itself to this configuration type. For example, the star configuration is appropriate for an altarpiece, but not for an entire Romanesque church built from stone.

The implementation of large WSNs, as required in this environment, requires the use of multi-hop approaches, dealing with several issues, such as routing topology control, *etc*. A lot of approaches have been proposed [1]. As an option, node clustering has been addressed by many researchers as a new technique that will allow for simpler topology management and improved network lifetime [1]. Previous studies have shown that organization of nodes into clusters provides greater energy efficiency [2]. Furthermore, several applications of wireless sensor networks require only an aggregate value to be reported to the operator [3]. In this case, the data gathered from each node is processed locally and aggregated at a coordinator node named cluster head (CH) and the redundant data (if any) is removed to provide more accurate reports about the local region being monitored, reducing the communication overhead.

There are different approaches for clustering algorithms. In homogeneous networks, where CHs are just regular sensor nodes, clustering algorithms must be distributed without coordination. In a few approaches, a centralized authority (the Sink) partitions the nodes offline and controls the cluster sizes according to the number of members, especially in heterogeneous networks where CHs are rich in resources.

The Low Energy Adaptive Clustering Hierarchy (LEACH) [4] is a clustering protocol that utilizes a random selection and frequent rotation of CHs for distribution of the total load across all nodes. The clustering process involves one iteration, after which a node decides whether to become a CH or not, with nodes alternately acting as CHs. Data communication in LEACH is based on single-hop communication model. There are two variants of LEACH, which are referred to as LEACH-C (LEACH-centralized), and LEACH-F (LEACH with Fixed clusters).

The Hybrid Energy-Efficient Distributed clustering protocol (HEED) [5] selects CHs through one shot (O(1)) time iteration according to a hybrid of the residual energy of nodes and another parameter such as node proximity to its neighbors or node degree. That is to say, HEED considers both energy and communication cost when selecting CHs. Unlike LEACH, the probability that two nodes within each other’s transmission range becoming CHs is small which means that the CHs are well distributed.

The Energy-Efficient Unequal Clustering (EEUC) [6] partitions the network into clusters of unequal sizes where the clusters closer to the Sink have smaller sizes than those farther away from the Sink. Thus, CHs closer to the Sink, can save some energy for the data relaying. Unlike others protocols such as LEACH and EEUC uses an energy-aware multi-hop routing protocol for inter-cluster communication, however, the setup phase in EEUC has a lot of overhead and as a result it consumes more energy in the setup phase when compared to LEACH. In stable state phase, EEUC saves energy by using an inter-cluster multi-hop data routing mechanism.

Energy-Efficient Level-based and Time-based Clustering (EELTC) [7] is a hierarchical clustering algorithm with multi-hop communication that establishes unequal clusters with very low controlling overhead. In this protocol the network is divided into radial regions using a heuristic formula. The Sink calculates upper bound and lower bound of each level and it then broadcasts the results across the network via a ‘hello’ message. All sensors determine their level by receiving this message from the Sink. Based on its level and energy each node sets a time to start advertising itself in the network to form clusters. The algorithm shows good energy efficiency and even load distribution across the whole network. A modified version called EELTC-M is proposed in [8]. This modification builds upon the previously proposed algorithm EELTC; the lengths of levels are modeled as an optimization problem based on the energy saved for each cluster. This energy is the difference of energy used by cluster head when using single-hop *versus* multi-hop communication model. In this manner, the cluster in the next level expands its size to cover some extra nodes instead. A comparative simulation was performed and EELTC-M showed to have a longer network lifetime compared to both the previous version and the EEUC protocol.

The remainder of the article is organized as follows. We discuss the original star-based communication architecture and sensor networks as well as the original energy model in Section 2. After that we provide a detailed investigation of current requirements for a real WSN Deployment. In Section 4 we propose a new energy efficient, robust and scalable architecture for WSN that satisfies the real requirements studied in previous section. Section 5 explains the simulation setup and presents the simulation results. In Section 6 we present the practical application and the experimental results of the proposed architecture. We then conclude our article in Section 7.

## 2. Original Star-Based Configuration

### 2.1. Node Description

**Figure 1.**
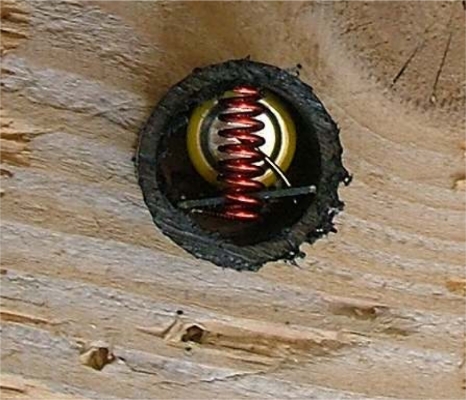
Node inserted in wood.

Figure 1 shows our leaf node inside a 2.5 × 5 cm cylinder embedded in wood. When installed, the node is powered and the hole sealed using a wood cap to maintain internal ambient conditions. This node, which uses a star wireless protocol, can last for more than 13 years, as shown in Section 2.3. Since this type of nodes only transmits measurements and it does not retransmit messages from other nodes, the energy required for RF is negligible when compared to other energy requirements, as proven in Section 2.2.

**Figure 2.**
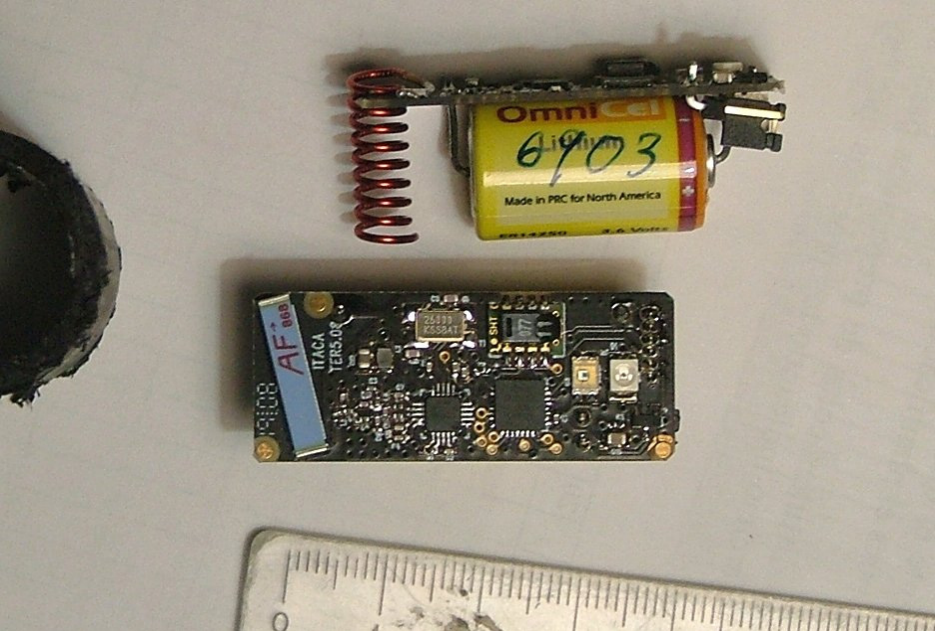
PCB component-side of the node and installed battery.

The original configuration is based on a set of these small nodes inserted in wood. These nodes collect ambient information and send a digest to a sink. Figure 2 shows an image of the node elements.

A Silabs C8051F930 low power microcontroller is the heart of the node. This relatively new 8-bit 8051 derivative performs really well and the available low power modes are very flexible. Power requirements specified on the datasheet were verified in this case as well. The node computes equilibrium moisture content of the wood based on the readings of ambient temperature and humidity using a Sensirion SHTx sensor.

The node also has an attractor for insects that are detected using light reflection variations produced with a high-efficiency LED from Avago and a high sensitivity sensor from Taos. This sensor is activated every 2 s. The node is powered using a high energy density 1,100 mAh, 3.6 V. lithium-thionyl battery.

The RF section of the node uses a Texas Instruments/Chipcon CC1101 ISM band transceiver. The chip has been configured for the 868 MHz European ISM band. This choice is a trade-off between small antenna size and range. The 2.4 GHz band has smaller antennas but it has shown poor performance with water moisture and crossing objects. The 433 MHz European ISM band is the best choice for our environment conditions, but the antennas are too large.

**Figure 3.**
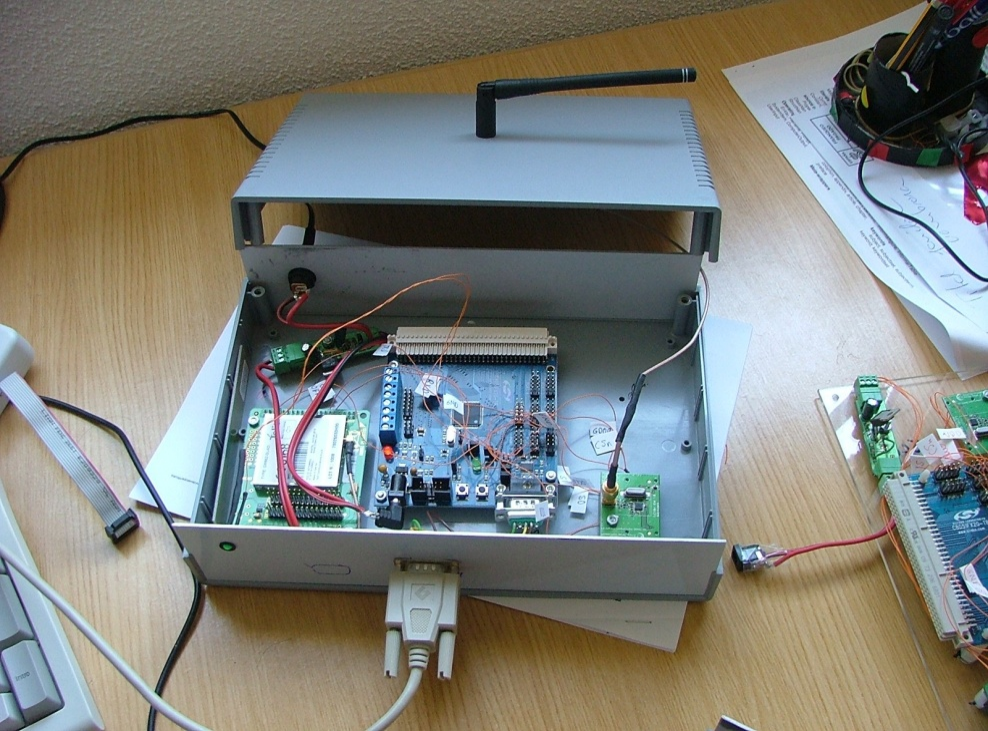
Internal arrangement of Sink components.

In the star configuration, nodes only transmit data, and the receiver part is not used. Data is sent three times per day. The time instant of transmission is calculated using a random number generator seeded with the node’s ID. Payload of the data packets can be reconstructed if some packets are lost, so such losses are not critical. The sink was built by assembling evaluation kits and commercial modules. Figure 3 shows an image of its internal arrangement.

In this case, the microcontroller that coordinates the entire system is a Silabs C8051F120 high performance microcontroller evaluation kit. This microcontroller can reach a peak performance of 100 MIPS. The RF radio modem section is based on a TI/Chipcon CC1101EMK868 evaluation kit. To this kit we attached a lambda/2 inverted dipole antenna from Antenna Factor.

**Figure 4.**
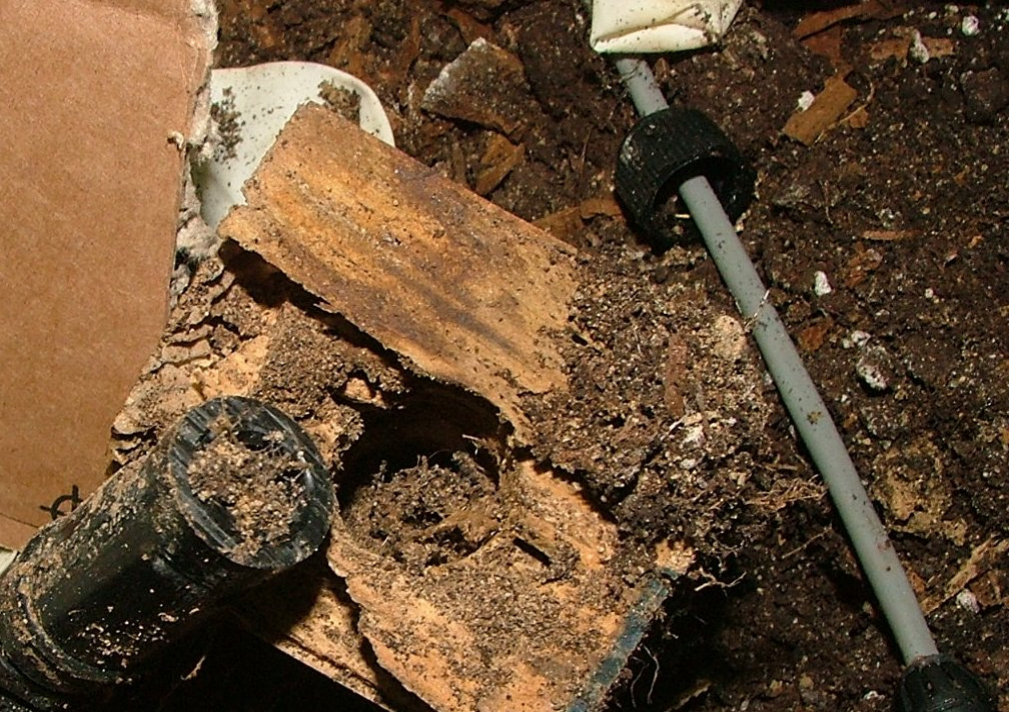
Node affected by termite activity.

**Figure 5.**
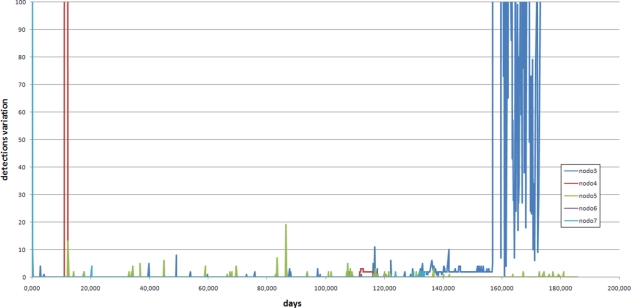
Number of termites detection during a 6 months experiment.

**Figure 6.**
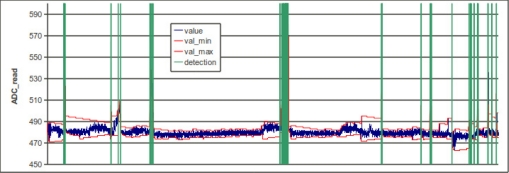
Instantaneous readings of the ADC converter and evolution of the detection algorithms.

The collected data was aggregated and sent using a GSM/GPRS Telit GM-862 to a remote location. A RS-232 serial link allows the configuration of the sink using any terminal emulation software on serial port such as Microsoft Windows HyperTerminal or Unix minicom. The collected data allows for continuous monitoring of the EMC of the wood and insect movement. As an example, Figures 4, 5 and 6 shows the results of a laboratory-controlled experiment using a colony of termites (*Reticulitermes lucifugus*), using nodes inserted in blocks of pine wood.

As a result of this experiment, we know that there is an exploratory phase and, sometimes, an abrupt increment in termite traffic until the node is affected by the termite activity. This forced us to develop an extra algorithm to detect the initialization of the insect sensing portion of the node and the resulting “silence” of the detector. Figure 5 shows an example of this situation.

To detect termites, we use an analog light detector and a LED. Every 2 s, the LED is turned on and the analog light detector if powered-up and read through an ADC converter of the microcontroller. Termite presence is not only factor which influences the readings, as temperature variations, dust and component aging also influence the readings, so we developed a detection algorithm that compensates for such variations and does not require factory adjustments. This algorithm works by recording the maximum and minimum value read, and triggering detection when the difference between both values is higher than a given threshold. The distance between both values is reduced automatically every N reads, so low speed variation phenomena are compensated automatically. Figure 6 shows an example of the behavior of the algorithm.

### 2.2. Node Energy Model

In a real implementation of a node it is necessary to consider all energy requirements, including energy requirements for sensors, microcontrollers, RF chip, *etc*. The energy requirements in Joules of each aspect of the node can be calculated using:

(1) E=I×V×t

where I is the current in Amperes, V is the voltage in Volts and t is the time in seconds. The complexity of the RF protocol will influence the requirements because it will involve higher processing requirements at the microcontroller level. Depending on the state of the node, the requirements of energy will be different. The total requirements of energy can be expressed as:

(2) $$E_{\mathrm{total}}=E_{\mathrm{rf}}+E_{\mathrm{protocol}}+E_{\mathrm{sensing}}$$

where *E_rf_* is the energy required by the radio modem portion of the node; *E_protocol_* is the energy needed to handle data to be transferred, the protocol overhead, and the radio modem signals handling (for instance, the communications signals between a microcontroller and independent radio modem); *E_sensing_* are the energy requirements for the sensing aspects and it is independent of the RF portion.

*E_rf_* is the only parameter that was considered when comparing WSN protocols and it is the one that was estimated in the simulations section.

The radio-modem has different energy requirements depending on whether it is transmitting, receiving, idle or sleeping. For example, the Texas Instruments/Chipcon CC1101 ISM band transceiver used in our implementation has four main states: idle, receiving, transmitting and shut-down. Table 1 summarizes some of the current requirements for the different states. Table 1 also includes real measured values. We also tested salient parameters for low power devices such as pin leakage that are not documented in datasheets.

For estimating the energy required for the radio modem E_rf_ we used the following equation:

(3) $$E_{\mathrm{rf}}=V\times \left(I_{\mathrm{sleep}}\times T_{\mathrm{sleep}}+I_{\mathrm{idle}}\times t_{\mathrm{idle}}+I_{\mathrm{rx}}+t_{\mathrm{rx}}+I_{\mathrm{tx}}\times t_{\mathrm{tx}}\right)$$

where the definition of each state is:

- Sleep: Radio modem in sleep-mode. This is the lowest possible power mode;
- Idle: Radio modem chip is ready to switch to receiving or transmitting mode. This mode requires significant energy because oscillator is running;
- Tx (transmission): RF data is being transmitted;
- Rx (reception): RF modem is in receiving mode. The energy requirements while receiving data and waiting for data are not significant.

The main objective of a low power protocol will be to maximize the time that the radio modem is in sleep mode. In real implementations, the energy requirement of each electronic part depends on its state. It is fundamental to test each part individually in different configurations in order to check whether the energy requirements specified on the datasheets are correct before considering its inclusion in the project (a mistake the authors have experienced first-hand in previous work). For the calculations, the maximum energy consumption must be considered.

### 2.3. Worst-Case Battery Life Estimation

The purpose of this section is to analyze the real energy required by the nodes. Although simulation results are excellent, this step allows us to demonstrate their validity in real situations. This analysis is based on a worst-case scenario to predict the life of the leaf nodes. Tested energy requirements showed life values that greatly exceeded the obtained results using these assumptions. In order to dimension the battery requirements, energy is measured in Ah (amperes × hour); this is the unit capacity for batteries. The original star configuration is the following:

- Three transmissions per day;
- One second per transmission, 10 dBm transmission power;
- No reception capability enabled.

The energy required for the node is reflected in Table 2. This table includes maximum energy for each part and time in each consumption mode.

Given these results, we can estimate the life of a given battery. For example, Table 3 shows battery life for the star configuration and different battery models.

For the battery originally installed in the nodes, and based on these results, we can guarantee a lifetime of 13 years for the star configuration.

## 3. Considerations for a Real WSN Deployment

There are many theoretical proposals for WSN which assume a homogeneous set of communicating nodes and whose main objective is to reduce the overall energy requirements and balance energy requisites between the nodes. Some assumptions of these designs are:

- The sink is fixed and located far from some of the sensors;
- All nodes in the network are homogeneous;
- Each node is limited to the same amount of available energy;
- The radio model assumes a symmetric channel where the energetic cost for a transmission from A to B is the same as for B to A;
- The RF “distance” is mainly based on physical distance.

The resulting protocol proposals represent a clear advance in this area, but are not applicable to our problem because their energy requirements are much greater, which drastically reduces the operative life.

Dealing with the implementation of the proposed system, our challenge was to obtain the benefits of multi-hop protocols without sacrificing leaf node life and maintaining the low cost of the network. To achieve our approximation to this ideal, we use these realistic assumptions for this particular application:

- Use of heterogeneous nodes;
- Possible use of supplementary energy sources on some nodes;
- The RF distance between nodes must be based on Link Quality Indicators (LQI);
- Application of an asymmetric radio model where the energetic cost for reaching A from B may be different than reaching B from A. This includes both reception and transmission.

### 3.1. Use of Heterogeneous Nodes

Provided that the life of the entire WSN can be enhanced, then specialization of a fraction of the nodes can considerably reduce the cost of the whole system. For example, a set of nodes can use larger antennas with better propagation patterns. This will allow our antenna constrained sensing nodes to be more easily reached, saving energy and employing fewer cluster heads. Better antennas also provide an additional benefit of a reduced hop-stretch.

An advantage of the heterogeneous approach is cost optimization, as the cost of the sensing nodes is minimized with the inclusion of better nodes. When a homogeneous configuration is proposed, we need to provide sufficient capabilities to all the nodes, increasing the unit cost of each node.

### 3.2. Supplementary Energy Sources

Compared to leaf nodes, cluster heads will require a significant amount of energy. This energy will be invested in inter-cluster communication and data aggregation. We believe that spending more energy on cluster heads is the best solution to save more energy in the leaf nodes.

Supplementary energy can be obtained using energy harvesting techniques (solar, thermal, vibration, *etc*.) that can provide an intermittent source of energy that can be stored [9]. If harvesting techniques cannot be applied, we can use larger batteries or a simple mains plug. As mentioned above, this node specialization can save money because the extra cost of the cluster head nodes can be largely compensated by the savings obtained from the leaf nodes.

### 3.3. Node Distance Metrics

Using only physical distance between nodes as metric is inadequate in their actual deployment; we believe that it can only be used for the purposes of model simulations to compare protocols. Our practical implementations of WSN showed that the best node distance metric is the Link Quality Indicator (LQI) that the RF chip provides when a data packet is received. If the selected RF chip does not offer this option, we recommend the use of another chip as money saved with an inferior chip may result in headaches in the future.

The LQI calculation depends on factors such as the modulation technique, the RF clock drift, the signal-to-noise ratio, the programmed bandwidth, *etc*. When more indicators can be obtained from the RF chip, better criteria can be applied. In short, the cluster construction must be based on LQI information. Geographical considerations will be used only in the deployment of cluster heads, but this deployment could be corrected using LQI aggregated data received in the sink.

## 4. EDETA Protocol Description

In this section we present the EDETA (Energy-efficient aDaptative hiErarchical and robusT Architecture) protocol. It is based on a two-level architecture type: the first level is made up of clusters, and the second by a dynamic tree. The clusters are selected randomly and the network structure is recalculated after certain number of rounds.

EDETA uses a calendar where events are scheduled based on wall-clock time. There are two kinds of nodes, cluster head (CH) nodes and leaf nodes (LF). Between cluster heads (CH), a tree structure is built to allow data communication to travel to a root sink. The protocol supports more than one sink in order to provide more scalability and some fault tolerant mechanisms.

In the proposed system, with fixed cluster nodes, which are specially located in order to be easily powered (by means of energy-harvesting subsystems or directly from a mains line), the cluster head nodes feature virtually “infinite” energy. The engineer has to carefully plan the deployment and choose where to put the CH nodes and which type of power supply they will have.

This approach allowed us to optimize the design of the leaf nodes, achieving a longer lasting, highly-competitive real WSN solution. EDETA’s operation is divided into phases, the initialization phase and the normal operation phase. The duration of each phase is time constrained. This way, EDETA can be used in applications that require bounded response times.

There are two variables that limit these phases’ duration. The TIME_CONFIG variable limits the initialization phase and the TIME_SUPERFRAME variable limits one round of the normal operation phase. The normal operation phase has a limited number of rounds defined by the parameter MAX_INTRAROUNDS. Therefore the normal operation phase will have a maximum duration of MAX_INTRAROUNDS × TIME_SUPERFRAME.

### 4.1. Initialization Phase

First of all, network initialization is performed. This phase lasts a maximum of two TIME_CONFIG. In this step, nodes select their CHs and a tree is built between CHs to send the collected data to the sink.

The exact instant for the initialization phase can be scheduled offline at a given calendar point, or can be forced by means of special signals. Both mechanisms are being studied. On one hand, the availability of a RTC clock in each leaf node allows the inclusion of a “first start” event at design phase. On the other hand, remote wake-up of nodes by means of radio signals is being considering. Although some prototypes of this mechanism are currently working, it was considered a bit risky to include them in the real system.

In this phase the network structure is built, consisting of three sub-phases. In the first part, with duration of half TIME_CONFIG, each node decides on its own if it is going to be a CH, based on the following procedure:

Every node calculates a probability threshold based on several parameters. Some of them are pre-defined in analysis time, such as the desired percentage of active CH’s in the network. Other change with time, such as remaining energy of the node, in comparison with the energy of the rest of the nodes in its environment, and the existence of nodes in the neighbor without access to any CH node.

By means of this threshold, every node will decide randomly if it must become a CH or remain as a leaf node. When a node decides to be a CH it sends HEAD messages to announce its role to the rest. Initially, the sink node is the only one to offer connectivity to the rest, and also publishes it by means of its HEAD message. At the same time, a CH starts receiving HEAD messages from the others and decides which CH it will join in order to send its data to the sink. At this level, a CSMA (Carrier Sense Multiple Access) protocol is utilized in order to try to avoid collisions, but collisions are still possible.

A CH will try to join another CH if the latter has established a path to the sink. That is, it can communicate with CHs that can reach the sink directly or through other CHs.

This procedure will finally build a routing tree, where the “root” is occupied by the Sink node. Each CH in the tree should have one “parent” CH node—which will route its messages to the Sink node—and a bounded set of “son” CH’s nodes, which deliver their messages through current CH node, Following with the “family” terminology, the “grandfather” CH node will be the “father” of a “father” node.

Meanwhile, nodes which will become leaf nodes, start receiving HEAD messages as well. They store them to decide which CH to join in the second part of this phase. The selection of the CH for these nodes is based on factors such as energy requirements and RF distance to CHs.

If a CH does not receive any HEAD messages, it sends a NEED_CH message. When a leaf node receives it, it reruns the procedure to decide whether it is going to be a CH or not, but with an increased probability of becoming a CH. In this case, the probability is increased in order to get the network to be built as fast as possible. This mechanism, along with the first random distribution of CHs, allows the protocol to rapidly adapt the CH population to the needs of the network. At the end of this sub-phase, the tree structure is built and leaf nodes have the necessary information to decide which clusters they are going to join.

In the second sub-phase, with duration of half TIME_CONFIG, leaf nodes start to join their selected clusters and the CH sends, in the response message, the time schedule in which each node has to send its data. After that, leaf nodes enter sleep state. A CH can only allow a limited number of nodes to join. This number is given by the parameters MaxSoft and MaxHard. A CH accepts all join request petitions until it reaches its MaxSoft. After that, it will only accept join petitions that have activated a last resort bit. When a CH reaches the MaxHard threshold, it will no longer allow new joins. This mechanism allows future incorporation of orphan leaf nodes and installation of new nodes without rebuilding the tree.

Finally, in the third sub-phase, with duration of one TIME_CONFIG, each CH in the tree sends to its father the amount of time needed to collect all the data from its nodes. The CH collects all this information and decides the time schedule in which its sons can send it the data. After that, the father repeats the process with its own father, sending the amount of time needed to collect all the data from its nodes and its sons; then, the grandfather decides the time schedule for data to be sent to it by its sons. This process continues until the entire tree schedule is completed.

### 4.2. Normal Operation Phase

When the tree is built, the normal operation phase begins. The network structure is complete and every node must send its data to the CH at the scheduled time, and during the remaining time the nodes enter the sleep state. When a CH has received the data from all its nodes, it aggregates it and sends it to its father at the established time.

As mentioned above, the father of a cluster informs on the time its sons will have to send their data. Sons will send their data only when they receive a POLL message. This allows the father to decide exactly when the data will be sent. This will require some fault tolerant mechanisms, as discussed in later sections, without inquiring collision of messages. Moreover, this polling mechanism allows timing between all the CHs in the tree to be synchronized.

This phase is performed during several rounds, with duration TIME_SUPERFRAME, as defined by the parameter MAX_INTRAROUNDS and planned in the calendar for each node. After that, the network structure is considered obsolete and every node restarts from the beginning at the initialization phase set out in the calendar.

### 4.3. Fault Tolerant Mechanisms

To guarantee the operation of the whole WSN it is important to provide it with some fault tolerant mechanisms. There are three main failing points: sinks, CHs and leaf nodes.

If a sink fails in a single-sink network, there is no possible immediate solution. But, as mentioned above, the protocol allows for the existence of multiple sinks in the network. In this scenario, if a sink fails, its tree becomes root-less. In this case, the CHs in the root-less tree will detect this situation because they do not receive information from its parent. They will then wake-up the surrounding CHs using a radio-triggered wake-up signal which is propagated by the other CHs in order to reconfigure the whole network. This mechanism requires extra energy and it is only implemented at CH level, not at LF node level. This radio-triggered wake up capability is proposed in [10].

Another important failure point exists at CH level. If a CH fails, all the nodes in the cluster are unable to send their data. In addition, its children CH’s in the tree will never reach the sink. To reduce the impact of this failure, the substitute node role has been introduced. A substitute node is a node within the cluster that shares the schedule with the CH and takes its place if it fails.

The substitute node is selected by the CH in the initialization phase, based on node’s energy and the proximity between them, but, in order to allow leaf nodes to remain in a low power state as long as possible, the leaf node will be notified during the first round of the normal operation phase.

This substitute node keeps monitoring the CH’s operation. This way, if a CH fails, its substitute node would detect that the CH is no longer responding to its messages, and it would take its place, acting as the new CH.

Another failure point exists at the leaf nodes level. If a leaf node temporarily malfunctions and performs a reset, it will become an orphan and must remain in “sniffer” receiving mode waiting for polling messages from other nodes in order to synchronize its clock and to receive information on the next initialization phase. Then the leaf node will remain in sleep mode until the initialization phase starts.

Depending on the configuration of the network, this solution can recover lost leaf nodes at the expense of its remaining energy.

## 5. Simulation Results

The experiments were performed with EDETA-e that we developed for the ns2.33 simulator [11] using the μAMPS extensions provided by MIT. These extensions are for ns2.1b5 and we ported them to ns2.33. In the following paragraphs the simulation parameters, scenarios, results and the experimental comparison of EDETA, LEACH (more extended clustering protocol) and Simple Star (ideal case) are shown and explained.

### 5.1. Parameters Used in the Simulations

In order to achieve realistic results we have used radio and consumption parameters extracted from actual implemented nodes in our laboratory [12]. The radio transceiver consumes 3.6 × 10^−6^ watts in sleep mode, 0.072 watts in receiving mode. The power consumed in transmission depends on the estimated distance to the receiver. Estimation is performed using the two ray ground model provided by the NS2.

The energy used by the radio circuit is 50 × 10^−9^ J/bit, the bandwidth 250 kbps and the carrier frequency 868 MHz. This carrier frequency allows nodes to transmit up to 200 m at maximum power.

The maximum transmission distance for a node is limited to 100 m with EDETA; there is no limit to transmission distance in either LEACH or Simple Star. No limits were placed in these two protocols because they assume that all nodes can reach the sink.

All the simulations were performed using an initial supply of 8 J of energy. The sink was located at the center of the scenario and nodes were randomly distributed. We performed simulations with 100 nodes in two random different scenarios of 200 × 200 m, 200 nodes in two different random scenarios of 500 × 500 m. Finally, a mean of all data were extracted.

To carry out LEACH simulations we used the implementation provided by the extensions mentioned above. And we implemented the star protocol, validated with empirical results. In LEACH, CHs are continuously retrieving data from their nodes and sending it to the sink. In star configuration, the nodes send their data to the sink every 20 s.

Simulation results for the EDETA algorithm were found using the same parameters as LEACH and Simple Star, except for the maximum distance a node can reach while it is transmitting its data. In EDETA it was limited to 100 m. The TIME_CONFIG parameter was set to 20 s for scenarios employing 100 nodes and 50 s for the scenarios employing 200 nodes. The TIME_SUPERFRAME parameter was set to 20 s.

### 5.2. Comparison between Algorithms

**Figure 7.**
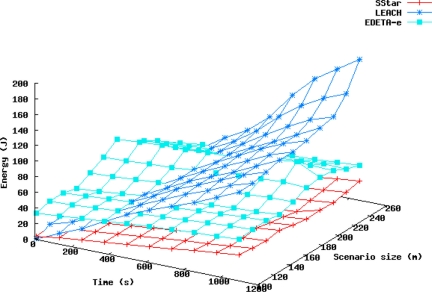
Energy consumption over the first 1,200 s for 100 nodes and areas from 100 to 260 m.

**Figure 8.**
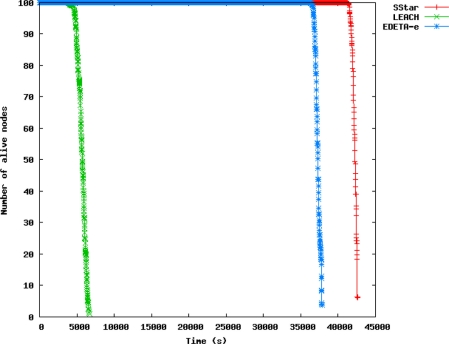
Live nodes for a population of 100 nodes in a scenario of 100 × 100 m.

Figure 7 represents the energy consumed is shown *vs.* time, and in Figure 8 the number of live nodes *vs*. time is shown, for all three protocols in the scenarios mentioned above.

As expected, neither the network diameter nor the number of nodes significantly affected the duration of the Simple Star protocol in these scenarios, as shown in Figure 7. In larger scenarios, however, the energy used to transmit to the sink would not be negligible and nodes that were far away from the sink would die sooner. Moreover, in practice, a network of say, 1 km^2^ would not be feasible using the Simple Star protocol because of its lack of scalability.

On the other hand, in the LEACH protocol, as the number of nodes increases, the number of CHs increases as well. The CH role was found to consume more power in this protocol.

In EDETA, however, as the number of CHs increases, the TIME_CONFIG parameter also increases in order to form the CHs tree to the sink. In the first half-TIME_CONFIG period, leaf nodes are awake and receiving from the radio channel, learning which CHs can be found around them; but in 10 s they enter sleep mode until they have to join their clusters, so there is a small overhead for leaf nodes at the beginning of the network formation but it is a price that must be paid in order to build the whole network structure. This can be seen in Figure 7, where EDETA starts with more energy consumption than the other two. But, after that, its growth is similar to Simple Star protocol.

These results can also be seen in Figure 8. Nodes in EDETA-e die faster than nodes in Simple Star, because of the energy that is consumed in network formation. However, the graphs show that the first node to die in EDETA-e does so 32,000 s later than in LEACH and the last node to die in EDETA only does so 1,700 s before Simple Star.

Therefore the simulations demonstrate that the techniques implemented in EDETA are effective in reducing energy consumption overall and in enhancing system lifetime. Our experiments show that EDETA can achieve reductions in energy consumption of up to factor of 8 when compared to LEACH. Furthermore the last node death in EDETA occurs over 10 times later than the last node death in LEACH. EDETA also provides added features that are more advanced, such as fault-tolerant mechanisms and bounded time [13–15].

## 6. Experimental Set-Up

A partial implementation of EDETA has been done in order to test the feasibility of the proposal and its real behavior.

### 6.1. Energy Requirements

The first step has been to estimate the real energy requirements of the leaf node for EDETA. For EDETA, the equivalent functional configuration used in the prototype is:

- Three transmissions per day;
- One second per transmission, 10 dBm transmission power;
- Two second mean time for CH polling (RX), maximum receiver sensitivity.

Table 4 summarizes maximum energy requirements for EDETA in this configuration.

Table 5 shows battery life for the EDETA configuration.

This life reduction for the EDETA configuration is perfectly acceptable in our case because it provides a higher level of flexibility, scalability and functionality.

### 6.2. EDETA-Based Implementation

The leaf nodes have been adapted by changing the firmware to activate the reception capability of the chip and by using a simplified protocol in which these nodes cannot be CH nodes. Some of the issues observed here will be addressed in future implementations.

**Figure 9.**
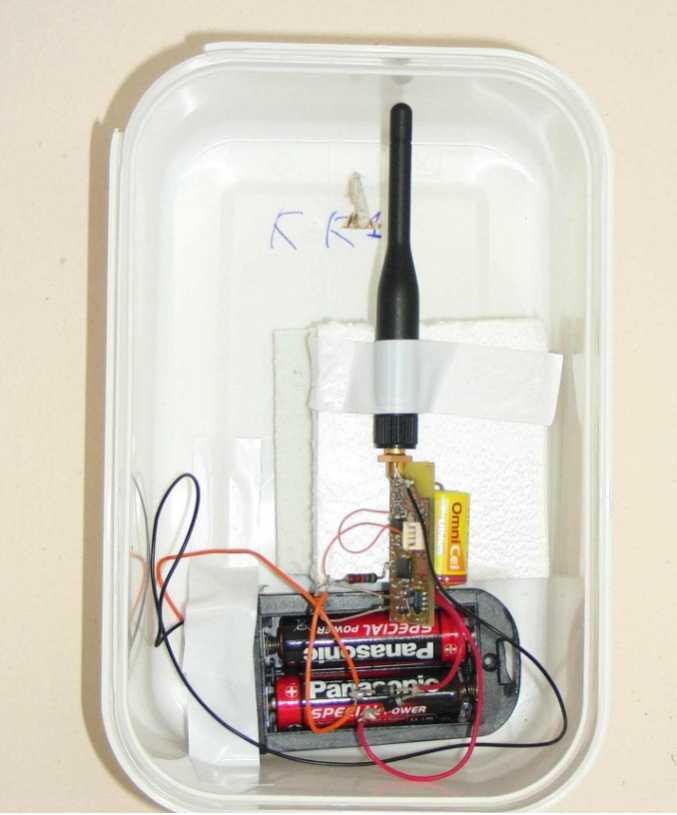
Aspect of the CH node prototype.

The CH nodes are the same nodes but have been provided with an extra energy reservoir and better antennas. For example, Figure 9 shows a node equipped with a lambda/4 monopole antenna, two alkaline AA 1.5 V batteries as an extra energy resource and two diodes to select the highest available voltage battery as the main source of energy. The ADC of the microcontroller can monitor the voltage of the extra energy source and send information about its state. If necessary, we can replace the batteries while the node is still in operation.

Thanks to the additional energy, specialized nodes are always selected for the CH role and the leaf nodes remain as simple sensors. No implementation changes are necessary. It is also possible, in a true full implementation, for leaf nodes to become CHs. The alkaline batteries utilized in this experiment are only for proof-of-concept testing. As mentioned above, an energy harvesting technique could provide the extra energy needed to operate the CH nodes.

### 6.3. Results

We conducted a set of experiments using three CH nodes and 12 leaf nodes. The configuration of the leaf nodes is the same as that proposed in the previous section. We forced a worst case tree creation scenario, where only one CH can communicate directly to the sink and the other CHs form a chain. This entails a distance of up to a 3-hop distance for a CH. The set-up has been working for two months and has not shown too many problems. No battery changes were required, including the CH nearest to the sink. The following issues arose from the experiments:

- TDMA de-synchronization: From time to time, CH lost access to some leaf nodes. We found that this occurred owing to an RTC inaccuracy that causes an incorrect timing scenario between the RX window timing instant in the leaf node and the TX polling time in the CH;
- Orphan nodes: Due to the time de-synchronization mentioned above, some spurious failures occurred, creating LF nodes with orphans.

These issues were patched and incorporated in the EDETA protocol described in Section 2. To solve the TDM de-synchronization, we concluded that events must be scheduled based on calendar events and not in time delays. If the RTC clock is set correctly, then we can ensure the correct operation of the whole WSN.

All timers are slightly corrected in each polling phase, and corrections are based on high precision clocks at the roots. Round-trip protocols for time synchronization can be considered in this implementation due to the waste of energy. In light of this, we decided to use a round-trip technique to estimate the delay, applying small local corrections in the nodes during normal operation in order to maintain the same temporary vision.

As this mechanism will be applied in all the nodes of the system, a correction wave of sorts will take place spreading from the root to leafs. To solve the issue of orphan nodes, we added a special event in the scheduling calendar for the recovery of orphans, by opening a (sufficiently long) temporary reception window that all the nodes can locate. This has been incorporated in EDETA as a fault recovering mechanism for leaf nodes.

## 7. Conclusions

This paper shows the real feasibility of an ultra-low energy, long life cluster-based WSN applied to environments such as heritage monitoring contexts where it is necessary to guarantee long life and no operator intervention. Novel node sensors designed for monitoring wooden masterpieces and historical buildings, in order to perform an early detection of pests, have been presented. These sensors have shown very satisfactory results in detection of termites and low power consumption.

We believe that this implementation reduces the gap between real star configurations and theoretical WSN models. The design of the protocol provides for implementation in low-cost low-power microcontrollers, reducing the overall cost of the WSN. Moreover, memory requirements are modest, including that required at the CH level, but, logically, are application specific.

The cost reduction is also optimized by the inclusion of a heterogeneous network, where we can greatly reduce the cost of leaf nodes at the expense of more costly CH nodes. Maybe the greatest difficulty that we found is the time and effort invested in the implementation of the CH on an 8051. This suggests that greater heterogeneity would be interesting, using a tiny OS at the CH and a larger microcontroller.

## Figures and Tables

**Table 1.** Current requirements measured and specified in datasheets. V_DD_ = 3.3 V.

**Parameter**	**Current according datasheet**	**Measured current**	**Units**

Receiving mode for 898 MHz (SRX)	15.7 (reduced mode)	19.6	mA
“SLEEP” mode (SPWD)	0.2	0.07	uA
Transmission mode, 868 MHz, FSK, 10 dBm	35.5	-	mA
Leakage current at pin CS (chip not selected)	Not documented	<1 μA	N/A
Current leakage at pin GD0	Not documented	0.1	mA

**Table 2.** Energy requirements for each part. Star configuration. V_DD_ = 3.3 V.

**Description**	**Current (μA)**	**Working time per day (s)**	**Annual requirements (mAh)**

Microcontroller sleep + RTC	1.0	86,313.60	8.75
Microcontroller active	3,000.0	86.40	26.28
Sensirion SHT1x active	900.0	5.00	0.45
Sensirion SHT1x unpowered	0.0		
Sensirion SHT1x sleep	0.3		
LED active	1,000.0	43.20	4.38
TAOS light sensor active	780.0	43.20	3.41
TAOS light sensor unpowered	0.0		
RF modem CC1101 transmitting + idle	35,000.0	3.00	10.64
RF modem CC1101 sleep	1.0	86,397.00	8.75
RF modem CC1101 receiving + idle	19.6	0.00	
		**Total energy required (mAh)**	62.68

**Table 3.** Estimated battery life. Star configuration.

**Battery model**	**Capacity (mAh)**	**Usable (%)**	**Estimated lifetime (years)**

EMB er14250 3.6 V (selected)	1,100	75	13.16
Lithium-thionyl 2/3 AA 3.6 V	1,700	75	20.34
Duracell 34 mm × 16.9 diam. 3 volts. DL12AB1 Ultra M3	1,500	75	17.95
Duracell Ultra 3 v. 27 × 15.6 diam DCLR2	950	75	11.37

**Table 4.** Energy requirements for each part. EDETA configuration. V_DD_ = 3.3 V.

**Description**	**Current (μA)**	**Working time per day (s)**	**Annual requirements (mAh)**

Microcontroller sleep + RTC	1.0	86,293.60	8.74
Microcontroller active	3,000.0	106.40	32.36
Sensirion SHT1x active	900.0	5.00	0.45
Sensirion SHT1x unpowered	0.0		
Sensirion SHT1x sleep	0.3		
LED active	1,000.0	43.20	4.38
TAOS light sensor active	780.0	43.20	3.41
TAOS light sensor unpowered	0.0		
RF modem CC1101 transmitting + idle	35,000.0	3.00	10.64
RF modem CC1101 sleep	1.0	86,397.00	8.75
RF modem CC1101 receiving + idle	19,600.0	6.00	11.92
		**Energy required (mAh)**	80.69

**Table 5.** Estimated battery life. EDETA configuration.

**Battery model**	**Capacity (mAh)**	**Usable (%)**	**Estimated lifetime (years)**

EMB er14250 3.6 V (selected)	1,100	75	10.22
Lithium-thionyl 2/3 AA 3.6 V	1,700	75	15.80
Duracell 34 mm × 16.9 diam. 3 volts. DL12AB1 Ultra M3	1,500	75	13.94
Duracell Ultra 3 v. 27 × 15.6 diam DCLR2	950	75	8.83
